# Failed Replication of Oxytocin Effects on Trust: The Envelope Task Case

**DOI:** 10.1371/journal.pone.0137000

**Published:** 2015-09-14

**Authors:** Anthony Lane, Moïra Mikolajczak, Evelyne Treinen, Dana Samson, Olivier Corneille, Philippe de Timary, Olivier Luminet

**Affiliations:** 1 Université catholique de Louvain, Department of Psychology, Louvain-La-Neuve, Belgium; 2 University of Denver, Department of Psychology, Denver, Colarado, United State of America; 3 Université catholique de Louvain, Department of Neurology and Psychiatry, Woluwe-Saint-Lambert, Belgium; University of Chicago, UNITED STATES

## Abstract

The neurohormone Oxytocin (OT) has been one of the most studied peptides in behavioral sciences over the past two decades. Many studies have suggested that OT could increase trusting behaviors. A previous study, based on the “Envelope Task” paradigm, where trust is assessed by the degree of openness of an envelope containing participant’s confidential information, showed that OT increases trusting behavior and reported one of the most powerful effects of OT on a behavioral variable. In this paper we present two failed replications of this effect, despite sufficient power to replicate the original large effect. The non-significant results of these two failed replications clearly exclude a large effect of OT on trust in this paradigm but are compatible with either a null effect of OT on trust, or a small effect, undetectable with small sample size (N = 95 and 61 in Study 1 and 2, respectively). Taken together, our results question the purported size of OT’s effect on trust and emphasize the need for replications.

## Introduction

The neurohormone Oxytocin (OT) has been one of the most studied peptides in behavioral science over the past two decades. Originally known for its role in labor and lactation, a growing body of evidence suggests that OT may also play a role in humans’ emotional and social lives. Research has for instance shown that OT increases trust [1], facilitates mind reading [2], makes people more sensitive to others’ feelings [3], promotes altruistic behaviors [4], is linked with parent-infant attachment [5] and enhances non-kin perceived trustworthiness and attractiveness [6].

Among these findings, the research on OT and trust has been both pioneering and the most prolific. In their seminal work, Kosfeld et al. [1] demonstrated that people who received intranasal OT were much more likely than those who received intranasal placebo to transfer money to a previously unknown partner during a dilemma known as the “trust game” (in the « Trust game », each participant assumes the role of an investor who can transfer money to a “trustee,” in whose hands the funds would triple. The trustee would then transfer all, some or none of the money, back to the investor). Using an adapted version of the same paradigm, Baumgartner et al. [7] showed that, after being betrayed by one person, people who received intranasal OT maintained their trust in other individuals, unlike people who received intranasal placebo who became suspicious of everyone. Building on these findings, Mikolajczak et al. [8] showed that OT increased trust with neutral or seemingly reliable partners, but that the trust-enhancing effect of OT disappeared when subjects were given subtle cues that the partner might be unreliable. Because the foregoing three studies had relied on an economic paradigm involving money transfer, Mikolajczak et al. [9] investigated whether OT would also increase trust when confidential information, rather than money was at stake. Using a “non-monetary” trust assessment paradigm, that we shall call the “Envelope Task” (see below), they showed that OT did increase trust toward strangers, thereby confirming Kostfeld [1] and Baumgartner’s [7] hypothesis that OT increases trust.

Compared to the “trust game” which involves money, the Envelope Task (ET) assesses participants’ trust that a stranger will not open an envelope containing confidential information about them. In this task, participants are first asked to fill in a questionnaire containing very intimate questions (i.e. about their sexual practices, including anal sex, sado-masochism, group sex, etc.) and to put the questionnaire into an envelope. When giving the instructions, the experimenter explains that he will not look at participants’ questionnaires, which will be read by an optical mark reader, handled by one of his colleagues, in order to preserve their intimacy and anonymity. The experimenter tells participants that they are free to close the envelope and, if they wish, to add sticky tape. So, in this paradigm, the level of confidence granted to a stranger (here the experimenter) is reflected in the degree of openness of the envelope 1 = Sealed and taped vs. 2 = Sealed vs. 3 = Left open (no participant taped the envelope without previously sealing it). Trustful people will tend to leave the envelope open (they trust the experimenter and think that he will not betray them), distrustful ones will be more likely to seal the envelope and even to add sticky tape (they do not trust the experimenter and think that he might betray them). In our first study using this paradigm [9], we obtained a very strong effect of OT on trust (Cohen's *d* based on ANOVA = 2.29; Cohen’s *d* = based on the ordinal regression = 3.07). Beyond its interest for the study of trust, the envelope test, or ET, seemed to be an ideal candidate as a manipulation check for ensuring that OT is active in a given study (and therefore helpful for interpreting null findings): it was short, easy to set up and powerful. These merits led us to use it as a manipulation check in a further study (Study 1). As explained below, the results were not those expected. This failed replication (despite sufficient power) led us to question the reliability of the ET paradigm. In order to clarify the discrepancies between the original findings and those of Study 1, we decided to replicate the paradigm for a second time (Study 2). Once again the results were very different from the original study, but very close to those of Study 1. As will be discussed later, these two failed replications are subject to different interpretations. But in any case, they confirm that this paradigm should not be used as a manipulation check.

## Material and Method

We will present the two studies in which we used the ET paradigm giving sufficient detail to enable readers to compare them to the original study. The first study aimed to determine whether OT influences mimetic desire and visual perspective taking, with the hypothesis that OT would increase mimetic desire and visual perspective taking. The second study examined whether OT influences compassion, with the hypothesis that OT would increase compassion.

### Ethics Statement

The following studies meet the guidelines for ethical conduct of research and were conducted in accordance with the Declaration of Helsinki. The Biomedical ethics committee of the Université catholique de Louvain approved the protocols. All subjects signed an informed consent where they were informed that they would receive either oxytocin or placebo. They were informed about possible adverse effects of oxytocin, the frequency of these adverse effects and about the potential allergic reaction to the product (and especially about how to recognize such reaction). Finally, they were informed of their duties (to report any symptom of allergic reaction) and rights (to stop the study whenever they wanted without providing any justification).

### Study 1: Oxytocin, Mimetic Desire & Visual Perspective Taking

#### Participants

95 healthy young adult males (*M*age = 22.53; SD = 2.89) took part in the study and were randomly assigned to receive either intranasal placebo (PL; n = 48) or OT (n = 47; 32 IU Syntocinon Spray – 4 puffs in each nostril–Fuerte Farmaceutica, Funchal, Portugal). The administration followed a double-blind procedure (the product samples were blinded with a sticky label by a colleague of the experimenter. This colleague linked each sample to a number written on the label and reported this Product–Number linkage on a list. The colleague kept the list until the end of the experiment). Exclusion criteria included medical or psychiatric conditions substance dependence and female gender. After providing written informed consent, participants were invited to complete several demographic and personality measures to ensure that both groups were equal regarding individual differences relevant to the study. These personality measures included alexithymia [10], the big five factors of personality [11], social desirability [12], empathy [13] and self-monitoring [14]. Each participant was paid 20€ and received a lottery ticket (1/95 chance to win) to win an extra 300€.

#### Procedure

Immediately after substance administration, participants were informed about the rest of the study and received the following instruction: after a 45-minutes break (during which all participants watched the same documentary: *“Lost Kingdoms of the Maya”*, National Geographic), they would take part in a computerized task (to assess mimetic desire, see [15]; to assess visual perspective taking; see [16]). Immediately after those tasks (lasting in total 20 minutes), they would find an envelope on their desk. This envelope will contain a questionnaire about the experimenters’ competence and is to be given to the experimenters’ supervisors. Participants were asked to fill in this questionnaire anonymously at the end of the experiment and the experimenter assured them that he would not look at their evaluation. However, participants were also told that they should feel free to seal the envelope and even add the sticky tape provided if they so wished (see detailed instructions in S1 Appendix). The nature of some questions aimed to ensure that participants would not wish to disclose the opinions they held about the experimenter (i.e. “*What are the experimenter’s 3 greatest flaws?*”). The ET took place 65 minutes after administration of the substances (placebo or Syntocinon Spray). This quite was similar to the original study (60 minutes) and is in accordance with the pharmacokinetic proprieties of vasopressin, OT’s closest molecule [17].

#### Statistics

The statistical analyses were run under SPSS 21 software. OT’s effect on trust was analyzed through a One-Way ANOVA with the condition (OT versus PL) as between-subject factor and with the degree of openness of the envelopes as dependent variable. We also performed an ordinal regression with condition (OT versus PL) as factor and with the degree of openness of the envelopes as dependent variable.

#### Results

**Fig 1:**
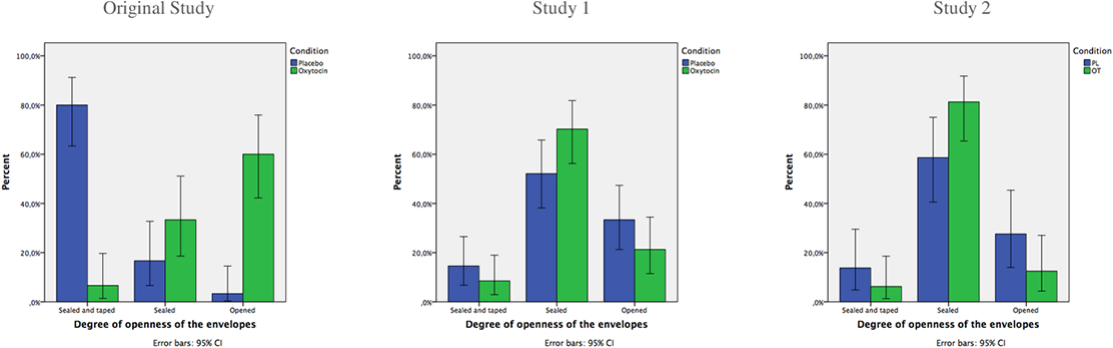
Percentage of participants displaying each behavior in the placebo and oxytocin condition for each study.

Preliminary analyses revealed no significant differences between groups regarding demographic and individual difference measured at baseline (all *p* > .124). We then performed a one-way ANOVA and an ordinal regression in order to compare the degree of openness of the envelopes between groups. No differences were found, either with the one-way ANOVA (*F*(1,93) = .229; *p* = .663) or with the ordinal regression (-2 Log-Likelihood = 17.801; *p* = .542). These findings suggest that OT does not enhance trust toward the experimenter (Fig 1). No moderation effects were found with the personality measures collected at baseline. By contrast, OT increased mimetic desire when two outliers (+2.5 SD) were removed (*F (1*,*90)* = 7.66, *p* < .007) but it had no effect on the visual perspective-taking task (all *F*-values for the main and interaction effects involving the OT factor < 1.5, all *p*-values > .23).

### Study 2: Oxytocin and Compassion

#### Participants

61 healthy young adult males (*M*age = 21.28; SD = 2.1) took part in the study and were randomly assigned to receive either the intranasal placebo (PL; n = 29) or OT (n = 32; 32 IU Syntocinon Spray – 4 puffs in each nostril–Fuerte Farmaceutica, Funchal, Portugal). The administration followed a double blind procedure (the blinding procedure was the same as the one reported in Study 1). Participants followed the same baseline procedure as in *Study 1*. They filled in questionnaires assessing the big five factors of personality [11], social desirability [12], empathy [13] and emotional dispositions [18]. Each participant was paid 15€ and received a lottery ticket (1/61 chance to win) to win an extra 500€.

#### Procedure

Immediately after substance administration, participants were informed about the rest of the study and received the following instruction: after a 45-minute break (during which participants watched the same documentary: *“Lost Kingdoms of the Maya”*, National Geographic), they would take part in a first computerized task (assessing compassion, adapted from [19]). At the end of this task (lasting 15 minutes) and before moving to another computer, they would have to fill in the questionnaire in the envelope on their desk. The experimenter warned them of the very intimate nature of the questions (about their sex life and fantasies) and assured them that he would not look at their questionnaires, which would be anonymously (participants were referred to by a code) transferred to the experimenter’s colleague who would process them using an optical reader device. However, participants were told that they were free to seal the envelope and even add the sticky tape provided if they so wished (see detailed instructions in S1 Appendix). This questionnaire investigates the subjects’ sexual practices and fantasies (see [9] for further details). The very intimate nature of the questions aims to ensure that participants would not want to disclose such information to a stranger. The ET took place 60 minutes after administration of the substances, in accordance with the pharmacokinetic proprieties of vasopressin, OT’s closest molecule [17].

#### Statistics

The statistical analyses were run under SPSS 21 software. OT’s effect on trust was analyzed through One-Way ANOVA with the condition (OT versus PL) as between-subject factor and with the degree of openness of the envelopes as dependent variable. We also performed an ordinal regression with condition (OT versus PL) as factor and with the degree of openness of the envelopes as a variable.

#### Results

Preliminary analyses revealed no significant differences between groups regarding demographic and individual difference measures at baseline (all *p* > .19). We then performed a one-way ANOVA and an ordinal regression to compare the degree of openness of envelopes between groups. No differences were found, either with the one-way ANOVA (*F*(1,59) = .295; *p* = .589) or with the ordinal regression (-2 Log-Likelihood = 15.726; *p* = .522). These findings suggest that OT does not enhance trust toward the experimenter (Fig 1). No moderation effects were found with the personality measures collected at baseline. Here, OT did not affect compassion (all *F*-values for the main and interaction effects involving OT < 1.511and all *p*-values > .224).

Table 1 summarizes and compares the relevant characteristics of the Envelope Task in both studies.

**Table 1:** Comparison of the relevant characteristics of the Envelope Task across the studies.

	General context of the study	Number of participant + Sex (OT/PL)	OT Dose (IU)	OT administration Mode	Location of the experiment	Number of experimenters	Sex of the experimenter who handle the Envelope Task	Content of the personal information	Effect of OT on the degree of envelope opening with One-way ANOVA	Effect of OT on the degree of envelope opening with ordinal regression	Means (SDs) for each groups*
Original study	Trust	60 males (30/30)	32	Single blind	Psychology Faculty Lab	1	Male	Sexual practices and fantasies	*F*(1,58) = 78.069, *p* < 0.001 (Cohen’s *d* = 2.29)	-2 Log-Likelihood = 11.57, *p* < .001 (Cohen’s *d* = 3.067)	OT = 2.53 (.63), PL = 1.23 (.50)
Study 1	Mimetic Desire	95 males (47/48)	32	Double Blind	University Hospital	2	Male	Assessment of the experimenters	*F*(1,93) = .229, *p* = .633 (Cohen’s *d* = -0.1)	-2 Log-Likelihood = 17.801, *p* = .542 (Cohen’s *d* = 0.125)	OT = 2.13 (.54), PL = 2.19 (.67)
Study 2	Compassion	61 males (32/29)	32	Double Blind	University Hospital	2	Female	Sexual practices and fantasies	*F*(1,59) = .295, *p* = .589 Cohen’s *d* = -0.15)	-2 Log-Likelihood = 15.726, *p* = .522 (Cohen’s *d* = 0.165)	OT = 2.06 (.435) PL = 2.14 (.64)

The original experimenter also performed the task in Study 1 and was present on site and supervised Study 2

* *The degree of openness of the envelope was coded as follows*: *1 = sealed and taped; 2 = sealed; 3 = left open*. *So the means here represent participants’ tendencies to trust the experimenter (closer to 3) or not (closer to 1)*.

## Discussion

In two studies (even if Study 1 is not a direct replication of the original study, as we did not use the same questionnaire, and has to be considered as a conceptual replication), we were unable to replicate our own findings on OT and trust. In addition to suggesting that the ET paradigm should not be used as manipulation check for OT effects, these (null) results show that nothing can be taken for granted about OT. The ET paradigm has seemed to lead to the strongest effect of OT on trust and we could not replicate that effect. This led us to look more closely at the literature about OT and trust. We noticed that Kosfeld’s [1] seminal finding was not always replicated either. Indeed, Baumgartner et al. [7] found no significant effect of OT on the *pre*-betrayal trust game. More recently, two independent studies also failed to replicate Kosfeld’s findings [20] [21]. This does not necessarily imply that the effect found by Kosfeld does not exist but rather that it should be interpreted more carefully. Nave and colleagues [22] computed the effect sizes of seven studies that have investigated the effect of intranasal OT on trust trough the trust game. Their analysis revealed that the combined effect size of intranasal OT on trust was small and not reliably different from zero (Cohen’s *d* = 0.077, 95%CI: [-0.124;0.278]).

In the case of the envelope task, we obtained a very powerful effect in our original study, followed by non-significant results in two studies with a sample size comparable to the original study. Five hypotheses may be raised to explain this; we shall examine them in turn. The first hypothesis suggests that OT’s effect on the ET paradigm exists and that the original study’s effect size is the “true” one. This hypothesis is however not likely as only 14 participants would have been needed to replicate such a large effect size (d = 2.29). Even when we take the lower bound of a large effect size regarding Cohen’s norms (i.e., *d* = 0.8), 84 participants should have been enough to replicate a large effect (we had 95 participants in Study 1). Therefore, we can exclude *a priori* the idea that our studies were underpowered to replicate a large effect. However, as shown below, it would have been underpowered to detect a small effect. The second hypothesis suggests that the effect of OT that we found in the original study does not exist and was purely the product of chance. Even with a *p* value < .000 (as in our original study), there is a chance (maybe one in a billion; we do not know the real *p* value as SPSS is limited to 3 decimals) of committing a type I error, namely of concluding to an effect that in fact does not exist. It would have been very unlucky but psychology is a probabilistic science, so we cannot formally exclude this hypothesis. Especially as, although intranasal OT is likely to cross the blood-brain barrier, only one study [23] has studied its exact pharmacokinetics. This study showed an elevation of CSF OT occurs 75 minutes after intranasal administration (whereas most of the behavioral tasks in the literature take place 45 minutes post-administration). However as this study relied on a very small sample (n = 11) the findings have to be handle cautiously, especially as no published studies have replicated these results since). Thenceforth, it is hazardous to conclude that it has formally been demonstrated that intranasal OT crosses the blood-brain barrier. Moreover, we cannot be sure that the usual doses used in the field (between 24 and 40 IU) can deliver enough OT to the brain in order to produce significant changes in individuals [24]. The third hypothesis suggests that the effect of OT that we found in the original study does not truly exist but that we artificially created it. In our original study, OT administration followed a single blind procedure. As demonstrated by Doyen et al. [25] the experimenter who knows the participants’ condition may unintentionally act differently so that participants’ behavior may be altered to confirm the researcher’s hypothesis. Because the original, single blind, study is the only one in which we obtained an effect of OT on the envelope task, we cannot formally exclude that the significant at the .05 level effect was the product of unconscious behavioral priming. The fourth hypothesis suggests that OT’s effect on trust in the envelope task does exist but it is far smaller than the one suggested by the original finding. When we pool the findings of Study 1 and Study 2 (which are very close to one another), we obtain an aggregated effect size of *d* = -0.12, [95% CI: -0.43;0.19]. If the “true” effect of OT on trust in the envelope task is not large (as suggested by the original study) but rather small, as suggested by the upper bound value of the CI in Study 1 & 2 (*d* = 0.19), we would have needed a sample of 1442 participants to render such a small effect significant. According to Simonsohn’s “small telescope approach” [26], the sample size required for achieving a 33% power for detecting the upper limit of the confidence interval in the replications was 258. Actually, Simonsohn’s approach also shows that even our original study would not have achieved sufficient power to meaningfully study the upper boundary of the effect found in the replication studies, as the original study’s power to detect a small effect was only 11%. Finally, our fifth hypothesis suggests that OT’s effect in this paradigm exists but that it goes in the opposite direction. The lower bound value (i.e., *d* = -.43) of the CI around the pooled effect size of Studies 1 and 2 indeed represents a small to moderate *negative* effect size. As unlikely as this hypothesis may sound, it has nevertheless received some support from Yao and colleagues [21] who recently showed that OT decreases trusting behavior in certain conditions (i.e. in women who have been betrayed).

As we write these lines, we do not know which hypothesis is true, although the first and last seem much less likely than the other three.

Accepting the second and third hypotheses (i.e., concluding that intranasal OT does not truly influence human behavior) implies that previously published effects of OT on behavior are all statistical artifacts. This is not impossible, as by setting p-values at .05, we conventionally accept a 5% false positive rate. Yet, this would imply a severe publication bias. Even though this is a chilling hypothesis, it is still plausible, and it was actually recently proven true for results concerning plasmatic measurements of OT [27]. Furthermore, a recent meta-analytic investigation on published studies involving intranasal administration of OT in humans [28] demonstrated that those studies are generally underpowered and report overestimated effects. This meta-analysis also demonstrated that the positive predictive value of those studies (using information on power, the pre-study odds and the alpha level) is low. Therefore, the authors suggested that most of the reported positive findings in this field are likely to be false positives.

Although it is possible that intranasal OT does not cross the brain-blood barrier and/or that it does not influence human behavior, it is also possible that OT has a small, yet real, effect on trust (our fourth hypothesis). If this hypothesis is true, it would raise two questions. First, does such a small effect warrant all the efforts and money invested in OT research? Second, if we admit that it is worth it, what are the moderators of OT effects? And, more particularly, can we predict/anticipate their effect? More and more research suggests that OT effects are context dependent (see [29] for a review). Regarding trust, it has been shown that OT effects are moderated by the characteristics of the subject (i.e. sex [21] and attachment type [30]) and by those of the target (i.e. its perceived level of reliability [8]). Note that these contextual effects of OT have not only been published regarding trusting behaviors but also regarding many other behaviors (i.e. [31; 32], see [29] for a review). If some “obvious” context parameters may influence OT’s action, some that are less obvious may also play a role, such as the sex or perceived reliability of the experimenter (which may induce a behavioral bias toward him or her), the location of the experiment (the faculty lab location may be warmer than the university hospital) or the general context in which an experiment takes place. In the present case, the original study was part of a broader study about the relation between trust and OT, so it cannot be formally excluded that the other trust paradigms did influence the results of the envelope task. If we accept that OT both enhances detection of social clues [33] and is context sensitive, it is possible that the variable preceding the ET did influence it, like a priming effect.

In any case, accepting the idea that intranasal OT can influence human behavior under some circumstances requires a theory that allows us to predict the exact circumstances in which OT does (or does not) do so. These failed replications show that many moderators of OT’s mechanism have yet to be discovered if we are to understand when OT influences human behavior and when it does not. Therefore, “fishing” practices leading to post-hoc identification of potential moderators should be avoided. Indeed, multiple hypothesis testing might increase the false discovery rate. If nonetheless a research provides an interesting post-hoc moderating effect, it should also report the other moderators that were tested but which did not yield a significant effect, as this would greatly help to identify which variables do or do not moderate OT’s effects. The reviewing process could encourage such disclosure.

Meanwhile, OT literature should be more open to non-significant results and keep in mind that paradigms that have worked once might not always maintain their promise. Whenever possible, a replication of the results should be attempted before publication and in any case OT literature should promote failed replications. Replication studies add great value to science [34; 35; 36] and are crucially needed in the OT research field if we want to dispose of a solid theoretical background for interpreting the significant and non-significant effects of OT.

## Supporting Information

S1 Appendix Detailed instructions for the envelope task in Study 1 and 2, respectively. (DOCX)
