# Supplementary material for: Failed Replication of Oxytocin Effects on Trust: The Envelope Task Case
Source: PLoS One. 2015 Sep 14;10(9):e0137000. doi: 10.1371/journal.pone.0137000 (PMC4569325; doi:10.1371/journal.pone.0137000)
Supplement: S1 Appendix — (DOCX) [file pone.0137000.s001.docx]

S1 Appendix: Detailed instructions for the envelope task in Study 1 and 2, respectively.

The Envelope Task has always been performed after a computerized task.

For (replication) Study 1, the exact instructions were as follows: *“You will now perform a task on the computer. The instructions concerning this task will appear on screen but if you have any question, do not hesitate to ask. At the end of the computer test, you will have to fill a questionnaire that is in the envelope on your desk. As a part of my PhD evaluation, my supervisors wants to have some feedback about my competence during this experiment. Therefore, you will be asked to answer a questionnaire about my abilities. Please do not be afraid of being sincere in your answers, I will not look at your questionnaire, I swear it. Your feedback put together with all the participants’ feedbacks will allow my supervisors to make constructive comments that will help me to improve myself. It is of course anonymous. At the end of the experiment, I will bring them all the questionnaires. I will just ask you to put the questionnaire back in the envelope once it is completed. You may close the envelope at the end and, if you want, you may even add tape. There is a tape dispenser on your desk”. There is some examples of questions they were asked to answer: “Please, tell us what was the 3 most important strengths and the 3 most important weaknesses of the experimenter?” “Please rate on a 5-point Likert scale (1 = not at all 🡪 5 = absolutely) your opinion on the followings: Has the experimenter been able to answer your questions? Does the experimenter know what he is doing? Does the experimenter looks self confident?…).*

For the original study and Study 2, the exact instructions were the follows: *“You will now perform a task on the computer. The instruction concerning this task will appear on screen but if you have any question, do not hesitate. At the end of the computer test, you will have to fill a questionnaire that is in the envelope on your desk. As we want to examine if oxytocin has an influence on sexual practices and fantasies, do not be surprised by the intimate or awkward nature of the questions. Please answer as honestly as possible. You will not be judged. Also do not be afraid of being sincere in your answers, I will not look at your questionnaire, I swear it. It will be handled by one of the guy in charge of the optical reading device who will not be able to identify you (thanks to the coding system). At the end of the experiment, I will bring him all the questionnaires. I will just ask you to put the questionnaire back in the envelope once it is completed. You may close the envelope at the end and, if you want, you may even add tape. There is a tape dispenser on your desk”. There is some examples of questions they were asked to answer: “What was your wildest sex experiment ?”, “Are you satisfied with your sex life? Could you describe it? (frequency, quality,…)” Please report on a 7-point Likert scale (1 = not at all, it disgusts me 🡪 7 = very much, I really like) your willingness to be involved in the following sexual practices: using sex toys, doing a threesome, having sex in public, watch other people having sex, watch porn before or during a sexual intercourse,…”*
